# Supplementary material for: An open-source framework for large-scale, flexible evaluation of biomedical text mining systems
Source: J Biomed Discov Collab. 2008 Jan 29;3:1. doi: 10.1186/1747-5333-3-1 (PMC2276192; doi:10.1186/1747-5333-3-1)
Supplement: Additional file 1 — Evaluation platform source code. The additional file contains the software evaluation framework discussed in this paper. The most current release can be downloaded from SourceForge.net [1]. See the accompanying README file for instructions on installation and use. Also included in the distribution is a collection of UIMA wrappers for some commonly used BioNLP tools and annotated corpora. [file 1747-5333-3-1-S1.zip › bionlp_uima_repository-1.0.1/docs/api/src-html/edu/uchsc/ccp/uima/ae/nlp/tokenizer/LingPipePOSTagger_AE.html]

```
001    /*
002     *
003     * The Original Code is UCHSC UIMA.
004     *
005     * The Initial Developer of the Original Code is University of Colorado.  
006     * Copyright (C) 2005.  All Rights Reserved.
007     *
008     * UCHSC Utilities was developed by the Center for Computational Pharmacology
009     * (http://compbio.uchsc.edu) at the University of Colorado Health 
010     *  Sciences Center School of Medicine with support from the National 
011     *  Library of Medicine.  
012     *
013     *
014     * Contributor(s):
015     *   William A. Baumgartner <william.baumgartner@uchsc.edu> (Original Author)
016     *   
017     * Created: November 2005
018     * Dependencies:
019     * TODO:
020     * Changes:
021     *          06/22/07 - wab - Added functionality to detect sentences and parse them if they exist. Otherwise, the entire document text is parsed.
022     */
023    package edu.uchsc.ccp.uima.ae.nlp.tokenizer;
024    
025    import java.util.Iterator;
026    import java.util.List;
027    
028    import org.apache.uima.analysis_engine.ResultSpecification;
029    import org.apache.uima.analysis_engine.annotator.AnnotatorConfigurationException;
030    import org.apache.uima.analysis_engine.annotator.AnnotatorContext;
031    import org.apache.uima.analysis_engine.annotator.AnnotatorContextException;
032    import org.apache.uima.analysis_engine.annotator.AnnotatorInitializationException;
033    import org.apache.uima.analysis_engine.annotator.AnnotatorProcessException;
034    import org.apache.uima.analysis_engine.annotator.JTextAnnotator_ImplBase;
035    import org.apache.uima.jcas.JCas;
036    
037    import edu.uchsc.ccp.uima.annotation.CCPDocumentInformation;
038    import edu.uchsc.ccp.uima.annotation.syntactic.CCPSentenceAnnotation;
039    import edu.uchsc.ccp.uima.util.UIMASyntacticAnnotation_Util;
040    import edu.uchsc.ccp.uima.util.UIMA_Util;
041    import edu.uchsc.ccp.util.external.lingpipe.LingPipe_Util;
042    import edu.uchsc.ccp.util.nlp.annotation.TextAnnotation;
043    
044    /**
045     * 
046     * This class uses the LingPipe POS tagger to create CCPTokenAnnotations. If SentenceAnnotations are present, they are tagged sequentially, otherwise
047     * the entire document text is assumed to be a single sentence.
048     * 
049     * @author William A. Baumgartner, Jr.
050     * 
051     */
052    public class LingPipePOSTagger_AE extends JTextAnnotator_ImplBase {
053    
054        private final boolean DEBUG = false;
055    
056        public static final String PARAM_LINGPIPE_MODEL_FILE = "LingPipeModelFile";
057    
058        /**
059         * Initializes the StanfordParser_AE by reading in the input parameters from the descriptor file and initializing a new StanfordParserUtil
060         */
061        @Override
062        public void initialize(AnnotatorContext ac) throws AnnotatorInitializationException, AnnotatorConfigurationException {
063            // get input parameters from the descriptor file
064            String lingPipeModelFile;
065            try {
066                lingPipeModelFile = (String) ac.getConfigParameterValue(PARAM_LINGPIPE_MODEL_FILE);
067            } catch (AnnotatorContextException ace) {
068                ace.printStackTrace();
069                lingPipeModelFile = null;
070            }
071    
072            // initialize the LingPipe tagging system
073            try {
074                LingPipe_Util.initializePOSTaggingSystem(lingPipeModelFile);
075            } catch (Exception e) {
076                e.printStackTrace();
077            }
078    
079            super.initialize(ac);
080        }
081    
082        /**
083         * This method processes the document text, and incorporates into the CAS TokenAnnotations based on the output of the LingPipe POS Tagger
084         */
085    
086        public void process(JCas jcas, ResultSpecification arg1) throws AnnotatorProcessException {
087    
088            Iterator docInfoIter = jcas.getJFSIndexRepository().getAnnotationIndex(CCPDocumentInformation.type).iterator();
089            String docID = "-1";
090            if (docInfoIter.hasNext()) {
091                CCPDocumentInformation srcDocInfo = (CCPDocumentInformation) docInfoIter.next();
092                docID = srcDocInfo.getDocumentID();
093            }
094    
095            UIMA_Util uimaUtil = new UIMA_Util();
096    
097            /*
098             * Check to see if there are any Sentence annotations in this CAS. If there are, then parse each sentence individually. If there are not, then
099             * treat the document text as a single sentence and parse it.
100             */
101    
102            int numSentenceAnnotations = jcas.getJFSIndexRepository().getAnnotationIndex(CCPSentenceAnnotation.type).size();
103            if (numSentenceAnnotations > 0) {
104                Iterator sentIter = jcas.getJFSIndexRepository().getAnnotationIndex(CCPSentenceAnnotation.type).iterator();
105                while (sentIter.hasNext()) {
106                    Object possibleSent = sentIter.next();
107                    if (possibleSent instanceof CCPSentenceAnnotation) {
108                        CCPSentenceAnnotation ccpSentAnnot = (CCPSentenceAnnotation) possibleSent;
109                        String sentenceText = ccpSentAnnot.getCoveredText();
110                        int spanOffset = ccpSentAnnot.getBegin();
111                        /* parse the sentence */
112    //                    System.err.println("PARSING SENTENCE: " + sentenceText);
113                        List<TextAnnotation> annotations = LingPipe_Util.runPOSTagger(sentenceText, docID);
114                        /* correct the annotations by applying the spanOffset */
115                        for (TextAnnotation ta : annotations) {
116                            ta.setAnnotationSpanEnd(ta.getAnnotationSpanEnd() + spanOffset);
117                            ta.setAnnotationSpanStart(ta.getAnnotationSpanStart() + spanOffset);
118                        }
119                        /* add the TextAnnotations to the JCas */
120                        uimaUtil.putTextAnnotationsIntoJCas(jcas, annotations);
121                    } else {
122                        error("Expecting CCPSentenceAnnotation, but instead got: " + possibleSent.getClass().getName());
123                    }
124    
125                }
126            } else {
127    //            System.err.println("PARSING FULL DOCUMENT");
128                /* treat the entire document text as a single sentence */
129                /* get document text */
130                String docText = jcas.getDocumentText();
131                /* parse the text, get back an ArrayList of TextAnnotations */
132                List<TextAnnotation> annotations = LingPipe_Util.runPOSTagger(docText, docID);
133                /* add the TextAnnotations to the JCas */
134                uimaUtil.putTextAnnotationsIntoJCas(jcas, annotations);
135            }
136    
137            /* Normalize syntactic tokens into TokenAnnotations, PhraseAnnotations, ClauseAnnotations, and SentenceAnnotations */
138            UIMASyntacticAnnotation_Util.normalizeSyntacticAnnotations(jcas);
139        }
140    
141        private void error(String message) {
142            System.err.println("ERROR -- LingPipePOSTagger_AE: " + message);
143        }
144    }
```
